# Supplementary material for: Reagent Effects on the Activated Partial Thromboplastin Time Clot Waveform Analysis: A Multi-Centre Study
Source: Diagnostics (Basel). 2023 Jul 22;13(14):2447. doi: 10.3390/diagnostics13142447 (PMC10377778; doi:10.3390/diagnostics13142447)
Supplement: Supplementary file 1 [file diagnostics-13-02447-s001.zip › diagnostics-2452237-supplementary.pdf]

Supplementary table: Site-specific age and gender means ± standard deviation (SD) of the CWA parameters from the reference population

REAGENT	PATHROMTIN SL						ACTIN FS						ACTIN FSL					
		SKH		SLGC		TOTAL		HSH		SH		TOTAL		NUH		SGH		TOTAL
Clot time, sec																		
Age	n		n		n		n		n		n		n		n		n	
<21	4	32.60 ± 5.01	33	34.20 ± 4.51	37	34.02 ± 4.52	13	26.55 ± 1.90	0	-	13	26.55 ± 1.90	5	29.54 ± 1.60	11	29.44 ± 2.43	16	29.47 ± 2.15
21 - 30	22	33.79 ± 3.43	99	33.99 ± 3.77	121	33.96 ± 3.69	21	24.92 ± 2.84	16	26.58 ± 1.92	37	25.64 ± 2.59	39	29.22 ± 1.56	94	29.03 ± 1.70	133	29.08 ± 1.66
31 - 40	29	34.16 ± 3.58	32	32.53 ± 3.46	61	33.31 ± 3.58	22	23.33 ± 1.88	8	27.09 ± 1.83	30	24.33 ± 2.50	59	28.94 ± 1.85	83	29.12 ± 1.91	142	29.04 ± 1.88
41 - 50	12	33.23 ± 4.32	0	-	12	33.23 ± 4.32	15	24.03 ± 2.21	18	26.57 ± 2.04	33	25.42 ± 2.45	16	28.89 ± 1.47	29	28.81 ± 2.16	45	28.84 ± 1.93
51 - 60	5	32.38 ± 6.96	1	-	6	31.98 ± 6.30	5	22.72 ± 2.03	1	-	6	22.88 ± 1.86	17	27.91 ± 1.97	16	28.50 ± 2.14	33	28.19 ± 2.04
>60	4	-	0	-	0	-		-	0	-	0	-	4	27.93 ± 1.02	9	27.83 ± 2.52	13	27.86 ± 2.12
Male	23	33.64 ± 4.38	81	33.78 ± 3.83	104	33.75 ± 3.93	34	25.45 ± 2.48	12	26.59 ± 1.31	46	25.75 ± 2.28	30	28.70 ± 1.92	96	29.00 ± 1.95	126	28.93 ± 1.94
Female	50	33.61 ± 3.78	86	33.73 ± 3.95	136	33.68 ± 3.88	43	23.65 ± 2.24	31	26.60 ± 2.17	74	24.89 ± 2.64	110	28.93 ± 1.71	156	28.90 ± 1.90	266	28.91 ± 1.82
Min 1, dOD sec^{-1}																		
<21	4	2.675 ± 0.854	33	2.644 ± 0.638	37	2.647 ± 0.650	13	3.715 ± 0.775	0	-	13	3.715 ± 0.775	5	4.019 ± 0.540	11	4.563 ± 1.153	16	4.393 ± 1.016
21 - 30	22	2.712 ± 0.564	99	2.813 ± 0.584	121	2.795 ± 0.579	21	4.225 ± 0.792	16	4.388 ± 0.660	37	4.295 ± 0.733	39	4.479 ± 1.063	94	4.556 ± 0.903	133	4.534 ± 0.949
31 - 40	29	2.899 ± 0.652	32	2.955 ± 0.620	61	2.928 ± 0.631	22	4.472 ± 0.752	8	4.514 ± 0.803	30	4.483 ± 0.752	59	4.863 ± 0.797	83	4.610 ± 0.797	142	4.715 ± 0.804
41 - 50	12	3.076 ± 0.486	0	-	12	3.076 ± 0.486	15	4.636 ± 0.433	18	4.664 ± 0.844	33	4.651 ± 0.679	16	5.011 ± 0.848	29	4.967 ± 0.738	45	4.983 ± 0.769
51 - 60	5	3.468 ± 0.367	1	-	6	3.415 ± 0.353	5	5.325 ± 0.848	1	-	6	5.457 ± 0.824	17	4.935 ± 0.607	16	5.375 ± 0.757	33	5.149 ± 0.709
>60	4	-	0	-	0	-		-	0	-	0	-	4	5.604 ± 1.259	9	5.241 ± 0.881	13	5.353 ± 0.972
Male	23	2.706 ± 0.505	81	2.594 ± 0.590	104	2.619 ± 0.572	34	4.090 ± 0.789	12	4.090 ± 0.709	46	4.090 ± 0.761	30	4.361 ± 0.936	96	4.433 ± 0.833	126	4.416 ± 0.855
Female	50	2.987 ± 0.639	86	2.996 ± 0.558	136	2.992 ± 0.587	43	4.557 ± 0.774	31	4.752 ± 0.752	74	4.639 ± 0.765	110	4.885 ± 0.863	156	4.856 ± 0.879	266	4.868 ± 0.871
Time of Min 1 (Tmin1), sec																		
<21	4	32.40 ± 4.92	33	34.05 ± 4.32	37	33.87 ± 4.35	13	26.44 ± 1.85	0	-	13	26.44 ± 1.85	5	29.22 ± 1.47	11	29.18 ± 2.23	16	29.19 ± 1.97
21 - 30	22	33.67 ± 3.33	99	33.89 ± 3.66	121	33.85 ± 3.59	21	24.88 ± 2.78	16	26.78 ± 1.84	37	25.70 ± 2.57	39	28.97 ± 1.48	94	28.77 ± 1.61	133	28.83 ± 1.57
31 - 40	29	34.00 ± 3.46	32	32.46 ± 3.36	61	33.19 ± 3.47	22	23.30 ± 1.82	8	26.94 ± 1.76	30	24.27 ± 2.41	59	28.69 ± 1.74	83	28.84 ± 1.80	142	28.78 ± 1.77
41 - 50	12	33.12 ± 4.22	0	-	12	33.12 ± 4.22	15	24.01 ± 2.17	18	26.12 ± 1.93	33	25.16 ± 2.27	16	28.63 ± 1.36	29	28.56 ± 2.04	45	28.58 ± 1.81
51 - 60	5	32.22 ± 6.74	1	-	6	31.87 ± 6.09	5	22.74 ± 2.06	1	-	6	22.90 ± 1.88	17	27.74 ± 1.88	16	28.23 ± 2.00	33	27.98 ± 1.92
>60	4	-	0	-	0	-		-	0	-	0	-	4	27.70 ± 1.00	9	27.67 ± 2.45	13	27.68 ± 2.06
Male	23	33.52 ± 04.24	81	33.71 ± 3.72	104	33.66 ± 3.82	34	25.38 ± 2.42	12	26.48 ± 1.23	46	25.67 ± 2.21	30	28.48 ± 1.81	96	28.75 ± 1.85	126	28.69 ± 1.84
Female	50	33.46 ± 3.69	86	33.60 ± 3.82	136	33.55 ± 3.76	43	23.63 ± 2.19	31	26.45 ± 2.10	74	24.81 ± 2.56	110	28.68 ± 1.61	156	28.64 ± 1.78	266	28.65 ± 1.71
Min 2, dOD^2 sec^{-2}																		
<21	4	0.444 ± 0.136	33	0.439 ± 0.103	37	0.439 ± 0.105	13	0.600 ± 0.123	0	-	13	0.600 ± 0.123	5	0.631 ± 0.082	11	0.723 ± 0.193	16	0.694 ± 0.169
21 - 30	22	0.452 ± 0.090	99	0.469 ± 0.097	121	0.466 ± 0.096	21	0.692 ± 0.135	16	0.708 ± 0.105	37	0.699 ± 0.121	39	0.712 ± 0.171	94	0.719 ± 0.146	133	0.717 ± 0.153
31 - 40	29	0.480 ± 0.106	32	0.496 ± 0.104	61	0.488 ± 0.105	22	0.741 ± 0.131	8	0.727 ± 0.130	30	0.737 ± 0.128	59	0.769 ± 0.126	83	0.726 ± 0.130	142	0.744 ± 0.130
41 - 50	12	0.515 ± 0.081	0	-	12	0.515 ± 0.081	15	0.766 ± 0.072	18	0.752 ± 0.132	33	0.758 ± 0.107	16	0.794 ± 0.122	29	0.790 ± 0.128	45	0.791 ± 0.125
51 - 60	5	0.581 ± 0.071	1	-	6	0.573 ± 0.067	5	0.884 ± 0.122	1	-	6	0.906 ± 0.122	17	0.796 ± 0.090	16	0.852 ± 0.134	33	0.823 ± 0.115
>60	4	-	0	-	0	-		-	0	-	0	-	4	0.905 ± 0.200	9	0.844 ± 0.148	13	0.863 ± 0.159
Male	23	0.449 ± 0.083	81	0.432 ± 0.099	104	0.436 ± 0.096	34	0.670 ± 0.133	12	0.667 ± 0.118	46	0.669 ± 0.128	30	0.700 ± 0.151	96	0.703 ± 0.138	126	0.702 ± 0.140
Female	50	0.498 ± 0.104	86	0.500 ± 0.092	136	0.499 ± 0.096	43	0.752 ± 0.132	31	0.764 ± 0.121	74	0.757 ± 0.127	110	0.774 ± 0.137	156	0.767 ± 0.145	266	0.770 ± 0.142
Time of Min 2 (Tmin2), sec																		
<21	4	28.58 ± 4.85	33	30.27 ± 4.17	37	30.08 ± 4.21	13	22.62 ± 1.74	0	-	13	22.62 ± 1.74	5	25.26 ± 1.31	11	25.24 ± 2.00	16	25.24 ± 1.77
21 - 30	22	29.90 ± 3.22	99	30.13 ± 3.52	121	30.09 ± 11.93	21	21.12 ± 2.67	16	22.98 ± 1.72	37	21.92 ± 2.46	39	25.08 ± 1.37	94	24.84 ± 1.48	133	24.91 ± 1.45
31 - 40	29	30.21 ± 3.33	32	28.73 ± 10.64	61	29.43 ± 3.35	22	19.62 ± 1.68	8	23.11 ± 1.62	30	20.55 ± 2.27	59	24.76 ± 1.57	83	24.90 ± 1.65	142	24.84 ± 1.61
41 - 50	12	29.36 ± 4.10	0	-	12	29.36 ± 4.10	15	20.33 ± 2.10	18	22.28 ± 1.81	33	21.39 ± 2.15	16	24.73 ± 1.22	29	24.65 ± 1.83	45	24.68 ± 1.63
51 - 60	5	28.48 ± 6.50	1	-	6	28.13 ± 5.88	5	19.10 ± 1.91	1	-	6	19.25 ± 1.75	17	23.93 ± 1.73	16	24.31 ± 1.80	33	24.11 ± 1.74
>60	4	-	0	-	0	-		-	0	-	0	-	4	23.88 ± 0.96	9	23.84 ± 2.33	13	23.85 ± 1.96
Male	23	29.73 ± 4.09	81	29.95 ± 3.59	104	29.90 ± 3.68	34	21.64 ± 2.28	12	22.71 ± 1.12	46	21.92 ± 2.09	30	24.63 ± 1.63	96	24.83 ± 1.69	126	24.79 ± 1.67
Female	50	29.70 ± 3.57	86	29.84 ± 3.68	136	28.79 ± 3.63	43	19.92 ± 2.08	31	22.61 ± 1.98	74	21.05 ± 2.43	110	24.77 ± 1.47	156	24.70 ± 1.63	266	24.73 ± 1.56

Max 2, dOD^2 sec^{-2}																		
<21	4	0.344 ± 0.109	33	0.345 ± 0.085	37	0.345 ± 0.086	13	0.513 ± 0.107	0	-	13	0.513 ± 0.107	5	0.499 ± 0.064	11	0.586 ± 0.174	16	0.559 ± 0.152
21 - 30	22	0.357 ± 0.076	99	0.378 ± 0.084	121	0.374 ± 0.083	21	0.603 ± 0.123	16	0.614 ± 0.095	37	0.608 ± 0.111	39	0.582 ± 0.146	94	0.584 ± 0.124	133	0.584 ± 0.130
31 - 40	29	0.380 ± 0.090	32	0.403 ± 0.093	61	0.392 ± 0.091	22	0.656 ± 0.125	8	0.631 ± 0.121	30	0.649 ± 0.122	59	0.626 ± 0.108	83	0.591 ± 0.113	142	0.606 ± 0.112
41 - 50	12	0.415 ± 0.072	0	-	12	0.415 ± 0.072	15	0.677 ± 0.070	18	0.649 ± 0.118	33	0.662 ± 0.099	16	0.644 ± 0.098	29	0.648 ± 0.122	45	0.647 ± 0.113
51 - 60	5	0.472 ± 0.084	1	-	6	0.466 ± 0.076	5	0.793 ± 0.109	1	-	6	0.811 ± 0.108	17	0.664 ± 0.075	16	0.691 ± 0.122	33	0.677 ± 0.100
>60	4	-	0	-	0	-	0	-	0	-	0	-	4	0.741 ± 0.158	9	0.708 ± 0.128	13	0.718 ± 0.132
Male	23	0.354 ± 0.075	81	0.346 ± 0.087	104	0.348 ± 0.084	34	0.583 ± 0.125	12	0.579 ± 0.114	46	0.582 ± 0.121	30	0.576 ± 0.130	96	0.574 ± 0.120	126	0.574 ± 0.122
Female	50	0.396 ± 0.089	86	0.403 ± 0.080	136	0.401 ± 0.083	43	0.664 ± 0.124	31	0.661 ± 0.109	74	0.663 ± 0.117	110	0.631 ± 0.116	156	0.625 ± 0.127	266	0.628 ± 0.123
Time of Max 2 (Tmax2), sec																		
<21	4	36.13 ± 5.04	33	37.83 ± 4.58	37	37.64 ± 5.49	13	30.25 ± 1.98	0	-	13	30.25 ± 1.98	5	33.22 ± 1.67	11	33.12 ± 2.53	16	33.15 ± 2.24
21 - 30	22	37.41 ± 3.49	99	37.63 ± 3.86	121	37.59 ± 3.78	21	28.61 ± 2.90	16	30.62 ± 2.01	37	29.48 ± 2.71	39	32.87 ± 1.65	94	32.34 ± 1.79	133	32.75 ± 1.74
31 - 40	29	37.80 ±3.68	32	36.14 ± 3.52	61	36.93 ± 3.66	22	26.96 ± 1.96	8	30.79 ± 1.94	30	27.98 ± 2.58	59	32.62 ± 1.96	83	32.81 ± 2.01	142	32.73 ± 1.99
41 - 50	12	36.83 ± 4.39	0	-	12	36.83 ± 4.39	15	27.68 ± 2.28	18	29.95 ± 2.12	33	28.92 ± 2.45	16	32.52 ± 1.52	29	32.47 ± 2.29	45	32.48 ± 2.03
51 - 60	5	36.00 ± 7.05	1	-	6	35.62 ± 6.38	5	26.34 ± 2.18	1	-	6	26.50 ± 1.99	17	32.04 ± 2.80	16	32.16 ± 2.24	33	32.10 ± 2.50
>60	4	-	0	-	0	-		-	0	-	0	-	4	31.50 ± 1.02	9	31.48 ± 2.63	13	32.49 ± 2.20
Male	23	37.29 ± 4.47	81	37.42 ± 3.91	104	37.39 ± 4.02	34	29.12 ± 2.57	12	30.26 ± 1.43	46	29.42 ± 2.37	30	32.62 ± 2.39	96	32.67 ± 2.06	126	32.66 ± 2.13
Female	50	37.22 ± 3.85	86	37.34 ± 4.04	136	37.30 ± 3.95	43	27.31 ± 2.31	31	30.31 ± 2.28	74	28.56 ± 2.72	110	32.59 ± 1.79	156	32.58 ± 1.99	266	32.58 ± 1.91
